# Association of Body Iron Status with the Risk of Premature Acute Myocardial Infarction in a Pakistani Population

**DOI:** 10.1371/journal.pone.0067981

**Published:** 2013-06-28

**Authors:** Mohammad Perwaiz Iqbal, Naseema Mehboobali, Asal Khan Tareen, Mohsin Yakub, Saleem Perwaiz Iqbal, Khalida Iqbal, Ghulam Haider

**Affiliations:** 1 Department of Biological and Biomedical Sciences, Aga Khan University, Karachi, Pakistan; 2 Department of Biochemistry/Pathology, National Institute of Cardiovascular Disease Karachi, Pakistan; 3 Department of Community Health Sciences, Aga Khan University and Aga Khan Development Network eHealth Resource Center, Karachi, Pakistan; Innsbruck Medical University, Austria

## Abstract

**Background:**

Coronary artery disease is very common in Pakistani population. Some of the studies carried out on Western populations have shown a relationship between body iron status as determined by the ratio of concentrations of serum soluble transferrin receptor (sTfR) to ferritin and the risk of acute myocardial infarction (AMI). In order to investigate whether increased body iron status has any relationship with the risk of premature AMI in Pakistani population, a case-control study was carried out.

**Methodology/Principal findings:**

In this case-control study, 203 consecutive AMI patients [146 males and 57 females; age range 18–45 years] admitted to the National Institute for Cardiovascular Diseases, Karachi, were enrolled with informed consent. In addition, 205 healthy controls whose gender and age (within 3 years) matched the patients, and who had a similar socio-economic background were recruited. Fasting venous blood was obtained and assessed for plasma/serum folate, vitamin B12, homocysteine, total cholesterol, triglycerides, LDL-cholesterol, HDL-cholesterol, sTfR and ferritin and blood lead. It was found that serum concentration of ferritin and blood lead levels were significantly higher in AMI patients compared to their age and gender-matched healthy controls (p value <0.05), while the concentrations of vitamin B12 and HDL-cholesterol were significantly lower in AMI patients compared to controls (p value <0.01). The ratio of sTfR to ferritin was significantly lower in AMI patients compared to controls [mean±SD/median (IQR) values 84.7±295/28.9 (38.4) vs 255±836/49.4 (83.8), respectively; p value <0.001]. Compared with the highest quartile of sTfR/ferritin (low body iron status), the OR for the risk of AMI was 3.29(95% CI, 1.54–7.03) for the lowest quartile (quartile 1) when the model was adjusted for vitamin B12 and HDL-cholesterol (p value for trend <0.01).

**Conclusions/Significance:**

This study shows a positive association between total body iron status and risk of premature AMI in a Pakistani population.

## Introduction

Body iron because of its ability to induce oxidative stress has been considered to be contributing to the pathogenesis of coronary artery disease (CAD). A number of studies carried out to ascertain the relationship between body iron stores and the risk of CAD have yielded conflicting results. A systematic review of the literature on this subject revealed that five studies reported significant association between serum ferritin and coronary heart disease (CHD), while nine studies did not show any such relationship [1]. Most of these studies used serum ferritin as a marker of body iron stores. Determination of serum soluble transferrin receptor (sTfR) concentration along with serum ferritin provided a more reliable tool for estimation of body iron status. In this regard, the concentration ratio of sTfR to ferritin was considered to be “a state-of-the-art measurement of the body iron stores” [2]. This study carried out in Finland showed that sTfR to ferritin ratio was associated with initial acute myocardial infarction (AMI) [2].

CHD is the leading cause of death in the South Asian subcontinent [3]. The major factors contributing to the development of CHD at early age include dyslipidemia, physical inactivity, hyperhomocysteinemia and genetic, environmental and nutritional factors [3].

Few studies have been carried out to investigate the relationship of serum ferritin and risk of CHD in the South Asian region [4], [5]; however, none of them have focused on the entire spectrum of body iron stores employing the ratio of sTfR to ferritin and investigating its relationship with premature CAD. Therefore, the present study was undertaken to find out whether or not iron body stores are associated with the risk of premature AMI in a hospital-based Pakistani population. Since Pakistani urban population has been found to have high levels of blood lead (Pb) which is also associated with hyperhomocysteinemia [6], another objective of the study was to investigate if high blood Pb levels in this population would modulate the relationship (if any) between body iron stores and risk of premature AMI.

## Methods

### Ethics Statement

This study protocol was approved by the Ethics Review Committee of the Aga Khan University, and a prior written informed consent was obtained from all the study participants.

### Participants’ Enrollment

In this case-control study, 203 consecutive adult patients (146 males and 57 females; age range 18–45 years) admitted to the National Institute of Cardiovascular Diseases a public sector institution for treatment of cardiovascular diseases in Karachi, who met the inclusion criteria were enrolled with informed consent. The enrollment started on November 2, 2009 and ended on May 18, 2011. The criteria for premature AMI patients included both male and female who were less than 45 years old and had a confirmed diagnosis of AMI. Confirmation of diagnosis was based on WHO criteria of clinical history suggestive of myocardial ischemia; ECG indications of myocardial damage and elevation of biochemical markers (creatine kinase and troponin I). They had not taken B vitamins (B6, B12 and folic acid) and iron supplements for the last 4 months (self-report); had no history of malabsorption syndrome, cancer, liver disease or uremia; and were not pregnant. In addition, 205 gender and age (within 3 years)-matched healthy individuals from the personnel of the Aga Khan University and other healthcare institutions of Karachi, who belonged to a similar socio-economic strata were included in the study with informed consent as controls. These individuals also had no history of consuming B vitamins (B6, B12 and folic acid) and iron supplements during the last 4 months, or suffering from malabsorption syndrome, cancer, liver disease, uremia or diabetes mellitus; and were not pregnant. These control subjects were included after clinical examination and relevant biochemical data. Both patients and controls belonged to the low income group as 90% of cases and 83% of controls had monthly house-hold income less than US $ 150. Anthropometric measurements which included height and weight were carried out by a trained research associate.

### Blood Sampling and Measurement of Biomarkers

Fasting venous blood (10 ml) was collected equally in both heparinized and nonheparinized tubes within 24 hours of hospital admission. Measurement of blood Pb was carried out in whole blood on graphite furnace using Hitachi Z-8000 Atomic Absorption Spectrometer with Zeeman’s background correction at the laboratories of the Pakistan Council for Scientific and Industrial Research, Karachi, Pakistan. Determination of serum concentration of vitamin B12 was carried out using a radioassay [7], while plasma homocysteine concentration was examined using an immunoassay-based kit, following the manufacturer’s instructions (Abbott Laboratories Ltd; Pakistan). Determination of serum ferritin, sTfR, folate, total cholesterol, triglycerides, LDL-cholesterol and HDL-cholesterol was carried out using kits obtained from Roche Diagnostics, USA. The minimum concentrations of detection for serum/plasma ferritin, sTfR, folate, total cholesterol, HDL-cholesterol, LDL-cholesterol, triglycerides, vitamin B12 and homocysteine were 0.5 ng/mL, 0.068 µg/mL, 0.64 ng/mL, 9.7 mg/dL, 3.0 mg/dL, 3.9 mg/dL, 8.9 mg/dL, 50 pg/mL, and 4 µmol/L, respectively. The minimum level of detection of blood Pb in the above mentioned method was 1 µg/dL.

### Statistical Analysis

All statistical analyses were carried out using IBM Statistical Package for Social Sciences® (SPSS) software version 19 for Windows® (Apache Software Foundation, USA). Independent sample t test was used to compare mean±SD values of continuous variables (serum folate, vitamin B12, total cholesterol, LDL-cholesterol, HDL-cholesterol, triglycerides, blood Pb) between cases and controls. However, Mann-Whitney U test was used to compare the distributions of homocysteine, ferritin, sTfR and sTfR/ferritin because the data for these variables were not normally distributed. Similarly, analysis of variance (ANOVA) was used to compare the mean values of continuous variables across the sTfR/ferritin quartiles, while Kruskal-Wallis nonparametric test was applied for comparing the distributions of homocysteine, sTfR and ferritin.

Conditional logistic regression was applied to examine the risk of AMI and sTfR/ferritin. Pairwise matching resulted in 201 case-control pairs. The model was adjusted for vitamin B12, HDL-cholesterol and blood Pb. For studying the interactions between body iron status and classical risk factors for AMI such as diabetes mellitus and low HDL-cholesterol (levels less than 35 mg/dL), the model was adjusted for fasting serum glucose concentration >126 mg/dL and low HDL-cholesterol. A p value of <0.05 was considered significant.

## Results

Analysis of the data revealed that concentrations of serum vitamin B12, total cholesterol, LDL-cholesterol, HDL-cholesterol and triglycerides were significantly lower in AMI patients compared to age and gender-matched healthy controls (Table 1). However, serum ferritin levels and blood Pb levels were significantly higher in AMI patients as compared to controls (p value <0.001 and p value = 0.011, respectively). In addition, it was found that the ratio of sTfR to ferritin was significantly lower in AMI patients compared to healthy controls (p value <0.001, Table 1). Moreover, the ratio of sTfR to ferritin had a statistically significant correlation with serum HDL-cholesterol (r = 0.145; p value = 0.003). Table 2 shows serum/plasma concentrations of homocysteine, folate, vitamin B12, cholesterol, LDL-cholesterol, HDL-cholesterol, triglycerides, and blood Pb by quartiles of ratio of sTfR to ferritin. Significant dose-response relationship exists for mean values of serum HDL-cholesterol across quartiles of ratio of sTfR to ferritin (p value = 0.004). We also found some significant results for serum folate, total cholesterol and triglycerides; however, dose-response relationship was not observed for these biomarkers across quartiles of ratio of sTfR to ferritin.

**Table 1:** Demographic characteristics, anthropometric measurements, and concentrations of biomarkers in patients with premature AMI and healthy controls.

Characteristics*	Patients	Controls	p value**
	(*n* = 203)	(*n* = 205)	
**Gender, n(%)**			
**Males**	146(71.9)	148(72.2)	0.95
**Females**	57(28.1)	57(27.8)	0.95
**Age(Years)**	41.6±4.5/43.0(5.0)	41.3±4.7/42.0(5.0)	0.51
**BMI, kg/m^2^**	25.7±3.6/25.1(4.8)	25.99±4.9/25.5(6.2)	0.53
**Homocysteine, µmol/L**	23.2±17.4/18.6(9.5)	23.45±18.6/18.6(9.1)	0.78
**Serum folate, ng/mL**	6.92±3.77/5.7(4.6)	6.89±3.72/6.1(4.8)	0.94
**Serum vitamin B12, pg/mL**	315±188/276(230)	364±180/331(230)	0.007
**Blood lead, µg/dL**	16.3±6.94/14.6(9.5)	14.4±7.7/12.7(8.3)	0.011
**Ferritin, ng/mL**	158.7±136.7/128.9(141.9)	87.1±74.7/66.7(86.8)	<0.001
**sTfR, µg/mL**	3.70±1.70/3.20(1.50)	4.14±2.91/3.45(1.60)	0.6
**sTfR/Ferritin**	84.7±295/28.9(38.4)	255±836/49.4(83.8)	<0.001
**Total cholesterol, mg/dL**	152±46/150(63)	171±34/170(47)	<0.001
**Triglycerides, mg/dL**	125±58/114(70)	154±93/131(97)	<0.001
**LDL-cholesterol, mg/dL**	98±39/94(53)	105±33/103(47)	0.023
**HDL-cholesterol, mg/dL**	28.2±11.3/27.0(11.0)	38.0±8.0/37.0(11.0)	<0.001

* Values are mean±SD/median (IQR) except for gender which are presented as n(%).

** p value was obtained by comparing mean±SD values using Independent sample t test, however, Mann-Whitney U test was used to compare distributions for variables homocysteine, ferritin, sTfR and sTfR/ferritin.

BMI =  body mass index, sTfR =  soluble transferrin receptor.

**Table 2:** Serum/plasma concentrations of homocysteine, folate, vitamin B12, cholesterol, LDL-cholesterol, HDL-cholesterol, triglycerides and blood Pb by quartiles of ratio of sTfR to ferritin in Pakistani patients with premature AMI.

Characteristics*	Q1	Q2	Q3	Q4	p value**
**Homocysteine, µmol/L**	23.9±20.7/18.2(9.7)	22.0±14.5/18.2(10)	24.2±18.4/19.4(8.3)	23.0±15.8/18.4(402)	0.41
**Folate, ng/mL**	7.45±3.83/6.39(5.34)	6.48±3.56/5.62(4.17)	6.23±3.42/5.1(4.5)	7.50±4.02/6.84(5.04)	0.03
**Vitamin B12, pg/mL**	315±159/294(219)	324±181/300(255)	352±193/334(200)	372±203/334(248)	0.11
**Cholesterol, mg/dL**	155±39/152(64)	169±45/164(51)	166±40/168(52)	157±41/156(52)	0.026
**LDL-cholesterol, mg/dL**	98.3±35.1/91.0(55.1)	108.0±39.7/105.0(51.0)	104.6±35.2/105.2(47.9)	97.2±35.3/93.0(46.3)	0.11
**HDL-cholesterol, mg/dL**	30.2±8.4/29.0(12.0)	32.9±13.6/32.0(12.0)	33.9±10.4/34.5(11.3)	35.7±10.3/36.0(15.8)	0.004
**Triglycerides, mg/dL**	126.7±62.4/115(73.0)	158.7±95.4/129.0(89.0)	148.6±80.7/132.0(86.0)	126.1±66.5/111.0(69.0)	0.004
**Blood Pb, µg/dL**	16.8±8.6/13.7(11.9)	15.1±6.6/14.0(7.5)	14.5±6.4/13.5(8.8)	14.9±7.6/13.0(8.4)	0.14

* Values are means±SD/median (IQR).

** p value was based on ANOVA comparing means of quartiles; however, Kruskal-Wallis nonparametric test was applied for comparing the distributions for homocysteine,

sTfR =  soluble transferrin receptor.

In order to evaluate the risk of premature AMI across the quartiles of ratio of sTfR to ferritin, conditional logistic regression analysis was carried out while adjusting for vitamin B12 and HDL-cholesterol. Compared to quartile 4 (reference) with the highest ratio of sTfR to ferritin (low body iron stores), the risk of premature AMI increases with each low quartile and a clear cut dose-response relationship is observed from highest quartile (low body iron stores) to lowest quartile (high body iron stores) [OR, 3.29(95% CI, 1.54–7.03); p value for trend <0.01; Table 3]. The risk of AMI remained more than 3 fold in individuals in the lowest quartile of sTfR to ferritin when the model was also adjusted for blood Pb [p value for trend = 0.912; Table 3]. Since diabetes mellitus is a well-established risk factor for MI in South Asia [8], the above mentioned logistic regression model was also adjusted for individuals having serum glucose levels greater than 126 mg/dL. Even after this adjustment, the odds (2.47) of having premature AMI remained significant (p value for trend = 0.027; Table 3) with the lowest quartile. Similarly, low HDL-cholesterol (levels less than 35 mg/dL) is another major risk factor of AMI in Pakistani population [9]. When the interactions of low HDL-cholesterol and fasting serum glucose levels greater than 126 mg/dL with body iron status were examined, they were found to be nonsignificant in this regression model (p values for interactions = 0.41 and 0.46, respectively).

**Table 3:** Risk of premature AMI in Pakistani adults with quartiles of ratio of sTfR to ferritin.

	Q1	Q2	Q3	Q4^5^	p value for trend
**Crude** 1	4.01(2.11–7.61)**	2.70(1.40–5.12)**	1.20(0.64–2.25)	1	<0.01
**Adjusted** 2	3.29(1.54–7.03)**	3.08(1.38–6.89)**	1.21(0.57–2.57)	1	<0.01
**Adjusted** 3	3.26(1.52–7.00)**	3.10(1.39–6.94)**	1.21(0.57–2.57)	1	0.912
**Adjusted** 4	2.47(1.29–8.16)*	3.24(1.29–8.16)*	1.11(0.46–2.69)	1	0.027

1 Values are matched OR (95% CI) from conditional logistic regression.

2 Values are matched OR (95% CI) from conditional logistic regression adjusted for vitamin B12 and HDL-cholesterol.

3 Value are matched OR(95%CI) from conditional logistic regression adjusted for vitamin B12, HDL-cholesterol and blood Pb.

4 Values are matched OR (95% CI) from conditional logistic regression after adjusting for blood Pb, vitamin B12, HDL-cholesterol and fasting serum glucose levels >126 mg/dL.

5 Considering the hypothesis that risk of premature AMI would be more at low ratio of sTfR to ferritin, the quartile indicating the highest ratio was taken as reference.

* p value <0.05.

** p value<0.001.

## Discussion

The relationship between body iron stores and risk of CHD, first proposed by Sullivan in 1981 [10], remained ignored until the results of Kuopio Ischemic Heart Disease Risk study (KIHD), carried out in Finland, became known to the scientific community [11]. In this study, a linear association between serum ferritin concentration and the increased risk of heart attack was shown in men [11], [12]. A strong epidemiological evidence regarding the role of body iron stores in carotid atherogenesis in Austrian men and women provided further support to the Sullivan hypothesis [13]. In a study carried out in Rotterdam, elevated levels of serum ferritin were found to be associated with the increased risk of MI in elderly population [14]. In a case control study involving 145 Indian men, serum ferritin levels were found to be directly correlated with the increased risk of AMI [4]. However, there were several other studies which found no association between body iron stores and risk of CHD [15]. For example, van der A DL et al. and Sun et al. reported lack of association between serum ferritin and CHD in women [16], [17]. Similarly, Sempos et al., Knuiman et al. and Galan et al. found no association between serum levels of ferritin and risk of CHD in both men and women [18]–[20]. It is important to mention that most of the studies examining the relationship of body iron stores and the risk of CHD used serum ferritin as the clinical marker of body iron status [4], [11]–[14], [16]–[23], however, in inflammatory conditions such as AMI, serum ferritin levels have been known to be elevated with concomitant decrease in serum iron [24], therefore, ferritin alone may not be an authentic clinical biomarker for iron status in AMI. On the other hand, sTfR as an index of tissue iron needs would not be influenced by the acute phase response, and perhaps, would provide better assessment of body iron status along with serum ferritin as a marker of tissue iron stores [25]. A ratio of sTfR to ferritin would, therefore, be a more reliable biomarker for body iron status even in an inflammatory condition, and therefore this study investigates the relationship of body iron stores with the risk of premature AMI.

Premature CAD is quite common in Pakistani population. A study from Karachi revealed that 16% of all the patients with AMI admitted to the Aga Khan University Hospital between January 2000–December 2002 were below the age of 45 years and 93% of them were men [26]. Besides genetic predisposition to developing CAD [27], dietary patterns have also been shown to be associated with the risk of AMI in this population [28], [29]. Western dietary pattern involving high consumption of red meat has been found to be associated with increased risk of AMI [28]. Since red meat is one of the major sources of body iron, it is conceivable that high intake of red meat by Pakistani population could be one of the factors contributing to the high body iron status and thus increased risk of AMI.

Pb pollution is a problem in this part of the world [30]. Body iron status can influence Pb absorption [31]. Therefore, we also measured blood Pb levels in cases and controls. AMI patients in this study had significantly high levels of blood Pb compared to controls which is in line with previous reports showing association between blood Pb levels and increased risk of cardiovascular disease [32]. However, when we adjusted our regression model for blood Pb levels, the risk of AMI remained similar to the risk when the adjustment was made for vitamin B12 and HDL-cholesterol. This indicates that body iron status in this population is associated with the risk of AMI independent of blood Pb levels.

According to the INTERHEART Study, the risk of MI among South Asians increases 2.48 folds in presence of diabetes mellitus [8]. In the present study, even adjusting for those individuals having fasting serum glucose levels greater than 126 mg/dL (a cut off level for diagnosis of diabetes mellitus), the risk of premature AMI remained nearly 2.5 fold high in those having highest iron stores of the body compared to those with lowest body iron stores. This also suggests that high body iron could be an independent risk factor for premature CAD.

Ideally, our controls should have been selected from the same communities to which AMI patients belonged. We recruited most of our controls from the personnel of various healthcare centers in Karachi; however, every attempt was made to pick those with socio-economic background similar to that of AMI patients. Both cases and controls belonged to the low income group as 90% of patients and 83% of controls had monthly house-hold income less than US $ 150.

There could be several reasons for increased body iron status in Pakistani AMI patients. Ninety-nine percent of Karachi population is nonvegetarian [33], therefore high consumption of foods rich in iron such as red meat, liver, kidney, heart, shellfish, clams, oyster, eggs could be contributing to high iron stores [34]. Moreover, foods cooked in cast iron pots and regular exposure to tobacco smoke must also be adding to the body iron [35]. Although our questionnaire did have information about consumption of red meat, poultry, fish and eggs, it did not include information about consumption of other food items rich in iron such as, shellfish, clams, oysters, nuts, seeds, or the nature of cooking pots and the extent of an individual’s exposure to tobacco smoke. This could be regarded as one of the limitations of this study. Tuomainen et al. have emphasized to include the information about users and nonusers of aspirin and antioxidants in studies concerning role of body iron stores in CHD because supplementation of these drugs could antagonize the risk-increasing effect of high iron stores [2]. Lack of information about the use of antioxidant vitamins and aspirin in the study population could be considered as another limitation of this study. Mean levels of total cholesterol, triglycerides and LDL-cholesterol were significantly lower in the study patients compared to controls; probably due to anti-lipid medications. Absence of information about the use of these medications is yet another limitation of this study. All of the blood samples from the patients were obtained within 24 hours of hospital admission. Since ferritin levels begin to increase on day 2 following AMI [36], it would be reasonable to assume that increased serum ferritin levels in AMI patients reflect body iron stores without any significant contribution from acute phase response. Measurement of serum levels of acute phase reactants in AMI patients could have been helpful, however, determination of markers of inflammation was not part of the study protocol and this would also remain a limitation of the present study. Despite these limitations, the present study did show an association of body iron stores with the risk of premature AMI in a Pakistani population. While such an association has been shown in adult Finnish men up to the age of 60 years [2], our study shows this relationship in relatively younger people (less than 45 years old) including both men and women. Our results, however, are different from those reported by Sun et al. who have shown lack of association between excessive body iron stores and CHD in female registered nurses in USA aged 30–55 years [17]. Similarly, in a German population serum concentrations of sTfR and ferritin were not found to be associated with CAD [37]. On the basis of these observations, it can be deduced that the relationship of body iron status with CHD could vary from one population to another. More data are, however, needed to further elucidate this relationship, especially in South Asian population which has the highest known rate of CAD [38], [39]. The mechanism by which high iron body status could be contributing to increased risk of AMI merits some discussion. It could be due to increased oxidation of LDL-cholesterol [21], increased oxidative damage [40], the promotion of lipid peroxidation, enhanced reperfusion injury and atherogenic properties [13], increased proliferation of vascular smooth muscle cells [41], or it could be because of the combination of all these along with other risk factors for CHD such as hypertension, inflammation and hyperhomocysteinemia which are highly prevalent in Pakistani population [3], [42].

### Conclusions

In an age and gender-matched case control study, high body iron status (as determined by the ratio of concentrations of sTfR to ferritin) has been found to be associated with increased risk of premature AMI in a Pakistani population.
